# UniTope & TraCR: A Universal Tool to Tag, Enrich, and Track TCR-T Cells and Therapeutic Proteins

**DOI:** 10.3390/medsci14010018

**Published:** 2025-12-31

**Authors:** Kanuj Mishra, Barbara Lösch, Dolores J. Schendel

**Affiliations:** 1 Medigene Immunotherapies GmbH, Lochhamerstrasse. 11, 82152 Planegg, Germany; 2 Champalimaud Foundation, Av. Brasilia, 1400-038 Lisbon, Portugal

**Keywords:** T cell receptor, epitope tag, universal antibody, adoptive cell therapy, quality control, immunotherapy analytics

## Abstract

Background: Adoptive cell therapy using genetically engineered recombinant T cell receptors (rTCRs) expressed in T cells (TCR-T cell therapy) provides precision targeting of cancer cells expressing tumor-associated or tumor-specific antigens recognized by the rTCRs. Standardized analytical tools are lacking to easily quantify receptor expression. Methods: To overcome this hindrance, a universal tagging system (UniTope & TraCR) was designed consisting of a minimal peptide epitope (UniTope) inserted into the constant region of the rTCR α or β chain and a high-affinity monoclonal antibody (TraCR) specific to this tag. Detailed biophysical, biochemical, and functional assays were performed to evaluate rTCR expression, folding, pairing, and antigen recognition, as well as antibody performance, using the UniTope & TraCR System. Results: Tagged rTCRs were stably expressed in human T cells with surface densities comparable to untagged rTCRs. The TraCR antibody bound UniTope with nanomolar affinity and no detectable cross-reactivity was observed for endogenous proteins expressed by human cells of diverse origin, importantly, including T cells of the natural T cell repertoires of multiple human donors. Functional assays confirmed that UniTope-tagged rTCRs preserved their antigen-specific cytokine secretion and cytolytic activity upon antigen-specific stimulation. The UniTope & TraCR System enabled robust detection of rTCR-expressing T cells by flow cytometry, and rTCR protein expression by Western blot or immunoprecipitation, supporting the quantitative assessment of receptor copy number and structural integrity. Conclusions: The UniTope & TraCR System provides a modular, construct-agnostic platform for monitoring engineered rTCRs, integrated into TCR-T cell therapies currently in development.

## 1. Introduction

Adoptive transfer of T cells engineered with tumor-specific T cell receptors (TCR) has matured into clinically validated TCR-T therapy for cancers such as melanoma, synovial sarcoma, and hematologic malignancies [1,2,3]. Despite substantial progress, a persistent limitation in broad application of different rTCRs with cost-effective preparation of patient-individualized TCR-engineered T cells, is the absence of standardized methods to measure transgenic TCR expression, confirm correct α/β pairing of rTCRs, and compare constructs used to express engineered TCRs originating from different discovery pipelines [4,5]. Current assays employ antibodies specific for TCR variable domains, peptide–MHC (pMHC) multimers, or antibodies specific for separate tags (i.e., RQR8, LNGFR, or CD34) that require complex vector design [6]. But all of these approaches have limits. Antibodies specific for TCR variable domains are not available for all variable domains and bind not only to the rTCR of interest, but also to some endogenous TCRs. Therefore, background noise is inevitable in FACS staining, rTCR-T cell enrichment will contain contaminants, and in vivo tracking is impossible. Multimers of pHLA can bind to TCRs of other specificities, have a short half-life, and cannot be manufactured for all pHLA combinations. Separate tags are not necessarily co-expressed at 100% with the rTCR; additionally, they are not suitable for in vivo tracking, since these tags originate from human molecules (i.e., CD20 and CD34) expressed on other cell types. Therefore, complex double staining with a T cell-specific antibody (i.e., Vß-specific) and the tag-specific antibody is necessary.

A non-perturbing epitope tag that can be universally integrated directly in all rTCRs would simplify this process. The ideal tag would display the following properties: (1) be short and structurally innocuous, (2) be positioned in a region of the TCR that is solvent-exposed and functionally silent, (3) allow detection by a single high-affinity monoclonal antibody under native and denaturing conditions, and (4) introduce no immunogenic or cross-reactive liabilities.

**Figure 1:**
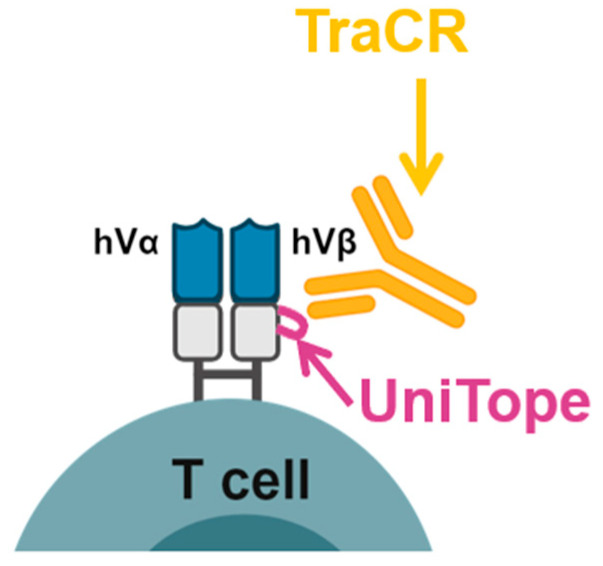
Overview of the UniTope & TraCR system for standardized detection of engineered T-cell receptors. Engineered α/β T-cell receptor (TCR) chains harbor a short epitope tag (UniTope) inserted into the constant region, which is recognized by a high-affinity monoclonal antibody (TraCR). The schematic illustrates the modular design and applications for flow cytometry, immunoblotting, and imaging.

Existing short tags that can be integrated into rTCR molecules, like the HIS-tag (six amino acids) or HA-tag (8aa), are likely to be immunogenic, so they are not appropriate for in vivo use [7]. To our knowledge, insertion sites in the constant region of an rTCR that change neither TCR functionality nor expression have not been identified previously. Insertion in the constant region is most suitable, since it can be applied to rTCRs of different specificity without changing their characteristics. To achieve these goals for tagging and tracking rTCRs, the UniTope & TraCR System was developed. In this system, a defined 6–12 amino acid (aa) motif (UniTope) is inserted into the constant region of either the α- or β-chain of an rTCR, flanked by flexible linkers to preserve tertiary structure. A corresponding monoclonal antibody (TraCR) recognizes this tag with high specificity. Together, they constitute a “plug-and-play” analytical interface enabling universal detection across diverse rTCR constructs (Figure 1). Based on three-dimensional (3D) modelling, it was hypothesized that UniTope insertion in an rTCR sequence would not disrupt TCR folding, pairing, or antigen recognition, and that TraCR would detect the tag in multiple assay formats.

Here, comprehensive validation of the UniTope & TraCR System is reported—from in silico design and alanine-scanning of antibody binding to functional testing in primary human T cells expressing tagged rTCRs. The findings demonstrate that the UniTope & TraCR System provides a robust, standardized method to quantify and compare engineered rTCRs in research and clinical development of TCR-T cell therapies.

## 2. Materials and Methods

The study was conducted in accordance with the guidelines of the Declaration of Helsinki and approved by the Ethics Committee of the Bavarian State Medical Association (BLÄK) (protocol code NCD-001, approval date: 18 May 2021).

### 2.1. Generation of Anti-UniTope (TraCR) Monoclonal Antibody

Lewis rats (*n* = 3) were immunized subcutaneously with a 12 aa synthetic peptide (DKGEVPKDRFSA) comprising the UniTope epitope. Booster immunization was made after three weeks, and hybridoma fusion was performed three days after the boost. Spleen cells from immunized rats were fused with the mouse myeloma cell line P3X63Ag8. More than 20,000 resulting hybridoma supernatants were subjected to rigorous sequential screening. Initial screening included ELISA to confirm specific binding to biotinylated immunization peptides, with counter-screening using irrelevant peptides to eliminate non-specific binders. ELISA-positive supernatants were then screened by flow cytometry for cell-surface binding to modified rTCRs expressed on TCR-deficient Jurkat 76 T cells, with specificity confirmed by lack of binding to TCR-negative Jurkat 76 T cells and to cells expressing non-tagged rTCR variants. An additional exclusion step ensured there was no binding to endogenous TCRs expressed by human peripheral blood mononuclear cells (PBMCs).

The monoclonal antibody (TraCR) was purified from hybridoma supernatant medium using the NAb Protein G Spin Kit (Thermo Scientific, Waltham, MA, USA), isotyped as IgG2a/κ, and sequenced by Rapidnovor Ltd. (Kitchener, Ontario, Canada). The aa sequences were back-translated into human codon-optimized nucleotide sequences. As the leader sequences are not resolved by protein sequencing, established leader peptides were incorporated for efficient expression [8]. The light and heavy chain nucleotide sequences were individually cloned into the pcDNA/3-TOPO mammalian expression vector (Thermo Fisher Scientific) and co-transfected into HEK293T cells to produce a recombinant antibody. The culture supernatant was collected 48 h after transfection and the monoclonal TraCR antibody was purified using Protein G chromatography.

### 2.2. Alanine-Scanning Dot Blot Analysis

To determine the critical aa residues within UniTope essential for TraCR antibody binding, alanine-scanning variants were employed. In this approach, a panel of UniTope peptides was synthesized, each featuring a systematic substitution of alanine at an individual position within the epitope sequence. Peptides were spotted onto PVDF membranes (Thermo Fisher Scientific) and incubated with TraCR hybridoma supernatant medium. Detection was performed using a horse radish peroxidase (HRP)-conjugated anti-IgG2a secondary antibody (Abcam, Cambridge, Great Britain). By comparing the intensity of antibody binding to the wildtype and alanine-substituted peptides, critical and non-critical amino acids for TraCR recognition were identified.

### 2.3. rTCR Constructs

Constructs encoding UniTope-tagged α or β chains, as well as the corresponding unaltered α or β chains of a rTCR sequence, were cloned into a retroviral vector (pES12.6-EF1α-TCRβ-P2A-TCRα) (BioNTech SE, Mainz, Germany) containing rTCRs specific for three different HLA-peptide complexes: New York esophageal squamous cell carcinoma-1 (NY-ESO-1:SLLMWITQC), preferentially expressed antigen in melanoma (PRAME:VLDGLDVLL), and Kirsten Rat Sarcoma viral oncogene homolog mutated antigen (KRASG12V:VVVGAVGVGK), presented by HLA-A*02:01, HLA-A*02:01, and HLA-A*11:01, respectively. Descriptions of the NY-ESO-1 (T11.8-10-17)-, PRAME-VLD (T4.8-1-29)-, and KRASG12V (T47.8-41-071)-specific rTCRs are published and have been submitted for patent protection [9,10,11]. All constructs were sequence-verified and validated for correct open reading frames.

### 2.4. Cell Culture and Retroviral Transduction of rTCRs

#### 2.4.1. CD8^+^ T Cell Isolation and Cultivation

Blood samples from healthy donors were collected after informed consent, in accordance with the Declaration of Helsinki and approval of the Ethics Committee of the Bavarian Chamber of Physicians. PBMCs were isolated using Ficoll (BioColl, Bio&SELL, Feucht/Nürnberg, Germany) gradient centrifugation. Human CD8^+^ T cells were enriched from PBMCs using negative immuno-magnetic separation (Dynabeads Untouched Human CD8 T Cells Kit, Invitrogen/Thermo Fisher Scientific) and were cultured in RPMI 1640 supplemented with 2 mM L-glutamine, 1 mM sodium pyruvate, 1% MEM non-essential amino acids (NEAA) (all Thermo Scientific), 10% human serum (Medigene Immunotherapies GmbH, Planegg, Germany), and 50 U IL-2 (Novartis, Basel, Switzerland). All cells were incubated at 37 °C and 5% CO_2_ under humidified conditions until use.

#### 2.4.2. Cell Line Cultivation

HEK293T cells (American Tissue Culture Collection (ATCC) CRL-1573) were cultured in DMEM supplemented with 2 mM L-glutamine, 1 mM sodium pyruvate, 1% MEM NEAA, and 10% fetal bovine serum (FBS) (Sigma-Aldrich, St. Louis, MO, USA). T2 (ATCC, CRL-1992) and Jurkat E6.1 TCR-deficient cells (courtesy of M. Heemskerk, Leiden, The Netherlands), also modified to express an NFAT-iEGFP switch and CD8 (unpublished observations) are designated hereafter as Jurkat biosensor cells. They were cultured in RPMI 1640 culture medium, supplemented with 10% FBS, 2 mM L-glutamine, 1 mM sodium pyruvate, and 1% MEM NEAA.

#### 2.4.3. Viral Transduction of CD8^+^ T and Jurkat Biosensor Cells with rTCRs

A gamma retroviral vector (pES12-6) containing the selected rTCR (specific for NY-ESO-1, PRAME, or KRAS G12V, respectively) or the same rTCR with different versions of the UniTope tag (6-12 aa flanked by GSG linkers) located at different positions in TRAC or TRBC were used to transduce Jurkat biosensor cells or human CD8^+^ T cells. Untransduced (UT) cells derived from the same cell line or healthy donors were cultured in parallel as controls.

In brief, HEK293FT cells were transfected with pcDNA3.1(+) packaging plasmid, encoding the transgenes gag and pol (BioNTech SE, Mainz, Germany) and Mirus transfection reagent (Mirus Bio LLC, Madison, WI, USA). PBMC-derived human CD8-selected T were activated with anti-CD3/anti-CD28-targeting beads (Dynabeads Human T-Activator CD3/CD28, Gibco Scientific/Thermo Fisher Scientific, Waltham, MA, USA) and 200 U/mL IL-2 (Proleukin-S, Clinigen, Yardley, Carlsbad, CA, USA) for 48 h. Jurkat biosensor cells or activated CD8 T cells were collected and transduced with the HEK293T-derived viral supernatant on retronectin (Takara Bio, San Jose, CA, USA)-coated plates at 32 °C via spinoculation. Transduced and UT cells were cultured 5 days before analysis.

### 2.5. Flow Cytometry

Cells were stained with PE-coupled TraCR antibody (2 µg/mL, 20 min, 4 °C) plus anti-CD8-APC (APC-Cy7 Mouse Anti-Human CD8, clone SK1, Becton Dickinson (BD), Heidelberg, Germany) in FACS buffer containing 1% BSA. As controls, the cells were stained with anti-TRBV antibodies specific for the respective TCR: anti-TRBV12-specific antibody for the NY-ESO-1 TCR (Beckman Coulter, Brea, CA, USA), anti-TRBV8-specific antibody for the PRAME TCR, and anti-TRBV5-5-specific antibody for the KRASG12V TCR (both Beckman Coulter, Krefeld, Germany). Data were acquired on an LSR-Fortessa flow cytometer (BD Biosciences, San Jose, CA, USA) and analyzed using FlowJo software (v10.8.1, BD Biosciences).

### 2.6. TCR-Activation Assay, Cytokine Secretion, and Cytotoxicity Assays

#### 2.6.1. Jurkat Biosensor TCR-Activation Assay

For functional analysis, Jurkat biosensor cells (NFAT-iEGFP, CD^8+^) were transduced with either the unaltered rTCR or the rTCR incorporating UniTope at the positions mentioned above. Jurkat biosensor cells, upon stimulation of the rTCR and subsequent triggering of the NFAT pathway, express eGFP in a dose-dependent manner. The Jurkat biosensor cells were co-cultured with T2-stimulating cells loaded with the relevant peptide (T2_Ld_rel_pep) or without peptide (T2 unl.), alongside controls using Jurkat biosensor cells alone as the negative control and PMA/Ionomycin stimulation as the positive control for complete activation. After 16 h, the eGFP signal was assessed via flow cytometry to quantify T cell activation.

#### 2.6.2. IFN-γ Release Assay

The assessment of IFN-γ release from UT or rTCR-engineered CD8^+^ T cells in co-culture with various target cells (T2 or K562), loaded or unloaded with relevant peptide, was made analyzing supernatant medium after 24 h of co-culture. The concentrations of cytokines were determined by ELISA (BD OptEIA Set Human IFN-γ, BD Biosciences). Optical density (OD) measurements were acquired using a Multiskan Microplate-Photometer (Thermo Fisher). To obtain precise results, background-corrected OD values were utilized, and data extrapolation was performed using a third-degree polynomial method.

#### 2.6.3. Live-Cell Imaging Cytotoxicity Assay

The assessment of target cell killing by TCR-T cells in ongoing co-culture assays was performed with time-lapse imaging using the IncuCyte S3 system (Sartorius, Göttingen, Germany), operated in accordance with the supplier’s protocols. Target cells were plated in either standard 96-well flat-bottom or ultra-low attachment round-bottom plates ahead of TCR-T cell addition. Cell lysis and cytotoxicity were tracked over time by scanning the plates at 4 h intervals. Tumor cell lines were genetically modified to stably express mKate2, a red fluorescent protein confined to the nucleus (IncuCyte NucLight Red Lentivirus reagent, Sartorius), enabling quantification of tumor cell depletion by measuring the loss of red fluorescence over time.

For healthy cell specimens which did not carry fluorescent markers, cytotoxic effects were evaluated qualitatively after 72 h using phase contrast images. These were independently reviewed by a panel of four trained scientists, who examined morphological changes, reductions in cell density, and evidence of induced T cell expansion as indicators of killing. Quantitative analysis was further conducted using phase contrast co-culture images collected up to 120 h and processed with the AI-based confluence mask provided in the IncuCyte S3 device with 2023A rev2 software (Satorius). Healthy cell confluency was measured and compared between TCR-transduced and UT T cell co-cultures. A threshold change of 20% relative confluency was established, based on observed variations in healthy target cells exposed to UT T cells; any reduction exceeding this benchmark was interpreted as significant cytotoxic activity mediated by TCR-T cells.

### 2.7. Statistical Analysis

Data were analyzed using GraphPad Prism version 9.0 (GraphPad Software, LLC, La Jolla, CA, USA). Results represent mean ± standard deviation of at least three replicate measurements. Comparisons between tagged and untagged rTCR groups were performed using an unpaired two-tailed Student’s *t*-test; *p* < 0.05 was considered significant.

## 3. Results

### 3.1. Design of UniTope and Insertion Sites in rTCR Constant Regions

The optimal epitope for universal tagging of rTCRs, or other human proteins, intended for therapeutic use, must have low immunogenicity to avoid immune detection in vivo in patients receiving TCR-T therapy employing tagged rTCRs. To achieve this property, we selected synthetic UniTope sequences that are closely related to human TCR beta variable (TRBV) regions, but lacking identity to any actual TRBV sequence to exclude cross-reactivity of TraCR antibody for natural TCRs and to minimize induction of antibodies against tagged rTCRs used in patient treatment. As the UniTope sequence is highly related to peptide sequences found in the extensive natural TCR repertoire, it is expected to be of low immunogenicity. The 12aa-specific (core 6 aa) epitope detected by TraCR was characterized using a variety of approaches.

**Figure 2:**
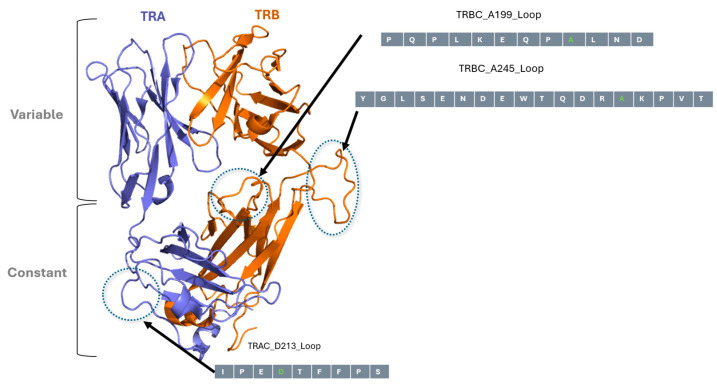
In silico modelled structure of NY-ESO-1 TCR and UniTope insertion sites. Three-dimensional model of the TCR α- and TCR β-chains of NY-ESO-1-specific TCR, highlighting the loops in constant regions targeted for UniTope insertion. The three loops selected for insertion of UniTope are depicted with the major aa positions used for insertion (light gray aa is the lead aa position).

Based on the crystal structure of the TCR (Protein Data Bank 6JXR, [https://www.rcsb.org/structure/6JXR, accessed on 28 September 2021]), 15 potential insertion sites were identified within the TCR α and β constant regions loops. Positions P198, A199, L200, and D202 (loop 1 of TRBC); A245, D238, and W240 (loop 2 of TRBC); and D213, I209, P211, and P214 (loop 3 of TRAC) were selected for analysis as UniTope insertion sites. Tags of varying length were flanked by flexible Gly-Ser linkers (GGGGS) to preserve structural integrity (Figure 2).

### 3.2. Epitope Mapping and Characterisation of Recombinant TraCR Antibody

**Figure 3:**
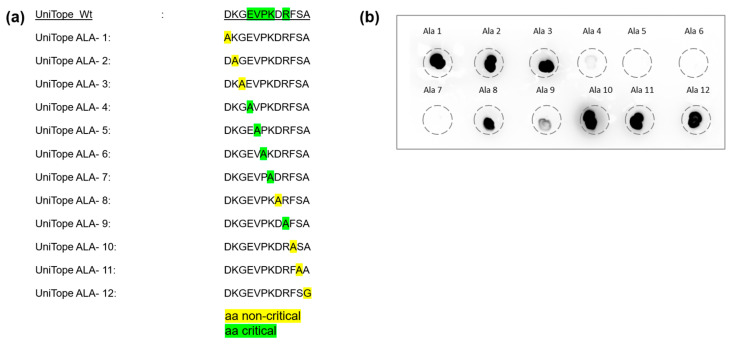
Alanine-scanning analysis defines the core epitope for TraCR recognition. (**a**) Wildtype and variant UniTope peptides employed for alanine scanning. (**b**) Dot blotting was performed to detect UniTope variants (ALA-1 to ALA-12) using TraCR hybridoma supernatant followed by detection with an HRP-labeled anti-IgG2a secondary antibody. Highlighted aa mark non-critical (yellow) and critical (green) aa for TraCR binding. Residues 4–9 are essential for recognition (>80% reduction upon substitution).

To determine the critical aa residues within UniTope essential for TraCR antibody binding, an alanine-scanning analysis was employed. In this approach, a panel of UniTope variant peptides was synthesized, each featuring a systematic substitution of an alanine residue at an individual position within the epitope sequence. Thus, each variant contained a single alanine residue replacing one native residue, enabling identification of key positions necessary for antibody interaction. The binding of the TraCR antibody to each variant peptide (UniTope ALA-1 to UniTope ALA-12) and the wildtype UniTope peptide was assessed in a dot blot assay. By comparing the intensity of antibody binding to the wildtype and alanine-substituted peptides, critical and non-critical aa for TraCR recognition were identified. These are highlighted in color (yellow: non-critical and green: critical) for visual interpretation (Figure 3).

This analysis demonstrated that positions 4 to 9 represent the main binding region for the TraCR hybridoma supernatant medium (Figure 3). Notably, alanine substitutions at positions 5 (Phe, F) and 7 (Lys, K) resulted in a greater than 90% reduction in binding signal, highlighting their critical contributions to the antibody–epitope interface and their central placement within the hypothesized 3D epitope pocket. These results enabled the definition of a minimal TraCR recognition motif, EVPKxR.

Due to concerns regarding the stability and batch-to-batch variability of hybridoma-derived antibodies, the TraCR antibody was recombinantly engineered for further characterization. The recombinant TraCR constructs were transiently expressed in HEK293T cells, and antibody-containing culture supernatants were harvested for subsequent analyses.

**Figure 4:**
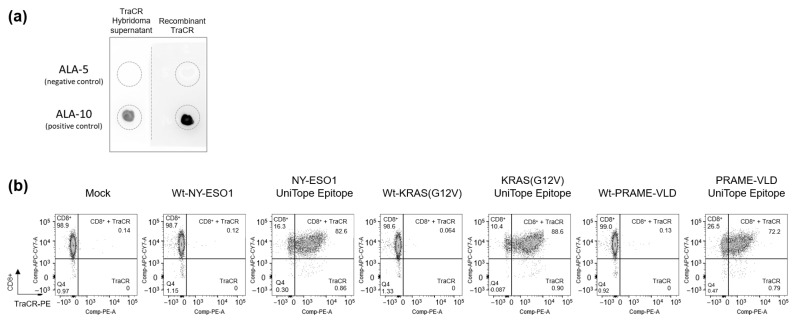
Characterization of the TraCR monoclonal antibody. (**a**) Dot blotting was performed to detect variant UniTope peptides (ALA-10 (positive control) and ALA-5 (negative control)) using a recombinantly produced TraCR primary antibody, followed by detection with an HRP-labeled anti-IgG2a secondary antibody. (**b**) Binding analysis of the recombinant monoclonal TraCR antibody to UniTope carrying NY-ESO-1-, PRAME-VLD-, or KRASG12V-specific rTCRs expressed in Jurkat biosensor cells. Secondary staining of the TraCR antibody was performed using an anti-rat IgG2a antibody labelled with PE.

Functional validation of the recombinant TraCR antibody was carried out using dot blot assays and flow cytometric staining. In dot blot experiments, the recombinant antibody recognized the ALA-10 peptide (positive control: DKGEVPKDRASA) but not the ALA-5 variant peptide (negative control: DKGEAPKDRFSA), recapitulating the specificity profile observed with the original hybridoma-derived TraCR antibody (Figure 4a). Further binding analysis revealed that the recombinant TraCR antibody specifically detected UniTope-tagged rTCRs in Jurkat biosensor cells, including NY-ESO-1-specific (T11.8-10-17), PRAME-VLD-specific (T4.8-1-29), and KRAS G12V-specific (T47.8-41-071) rTCRs carrying the UniTope tag at position A245 in loop 2 of the TRBC domain (Figure 4b). These experiments confirmed that the recombinant TraCR antibody retained the precise binding characteristics of the hybridoma-derived supernatant medium and is a reliable tool for detecting UniTope-tagged rTCRs.

### 3.3. Assessment of TraCR Cross-Reactivity for Natural Proteins

The core motive EVPKxR was used for a human proteome-wide search using BLAST+ 2.16.0 (https://blast.ncbi.nlm.nih.gov/Blast.cgi?PAGE=Proteins, 11 December 2025) to identify proteins carrying this epitope. By this approach, we could identify 43 proteins that carried the recognition motive of TraCR; 42 were intracellular proteins and therefore not accessible for the TraCR antibody. Only the sodium/potassium-transporting ATPase subunit beta-3 (ATP1B3) was expressed, according to UniProt release 2024_06 (https://www.uniprot.org/, 11 December 2025), on the plasma membrane of various cells, including blood lymphocytes, brain tissues, and brain microvascular endothelial cells [12,13,14]. To clarify if this epitope is accessible for TraCR binding and could potentially limit use of TraCR for in vivo tracking of TCR-T cells in the future, we cultivated human cells identified to express ATP1B3 and stained them with TraCR. No binding was detected, allowing for the conclusion that the epitope in this non-TCR protein was not accessible for TraCR binding (unpublished observations).

### 3.4. Localization of UniTope in rTCRs

**Figure 5:**
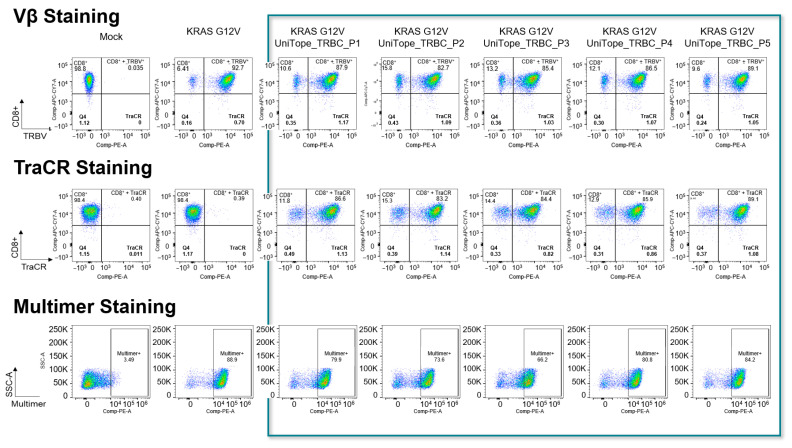
Flow-cytometric detection of UniTope-tagged rTCRs. FACS analysis of Jurkat biosensor cells expressing tagged or untagged rTCRs stained with fluorescent mAb specific for TRBV5-5 (upper panel) or TraCR (middle panel). To verify correct folding, the rTCRs were also stained with peptide-TCR-specific multimers (lower panel). The columns show mock transduced, untagged rTCRs as well as rTCRs tagged with UniTope at different positions (P1 = A199, P2 = W240, P3 = D202, P4 = A245, and P5 = I209 (TRAC)).

Flow-cytometric analysis demonstrated robust surface expression of UniTope-tagged α/β rTCR constructs in both Jurkat biosensor cells and primary human CD8^+^ T cells. To ensure that the constructs were properly expressed, folded, and functional, Jurkat biosensor cells were transduced with the corresponding UniTope-tagged KRASG12V-specific rTCR constructs and stained with TraCR and a monoclonal TRBV5-5-specific antibody, as the positive control. Several UniTope variants performed equally well with respect to expression and staining and were carried forward for further analysis (Figure 5). Staining with an HLA*11:01-KRASG12V multimer ensured correct folding of the tagged rTCR.

### 3.5. Tagged rTCRs Retain Functional Integrity

**Figure 6:**
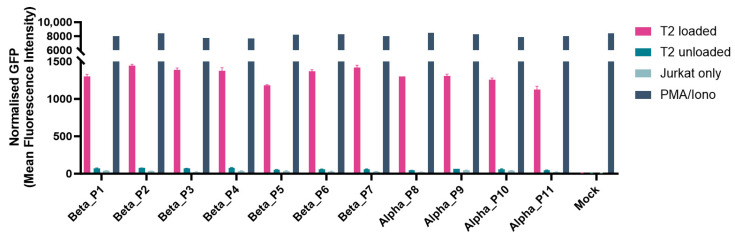
Functional analysis of different tag positions. Jurkat biosensor cells were co-cultured with T2 cells pulsed with (loaded) or without (unloaded) the relevant peptide, along with controls (negative control: rTCR-expressing Jurkat biosensor cells only; positive control for full activation: PMA/Ionomycin). After 24 h, the eGFP signal was analyzed by flow cytometry as the read-out for rTCR activation. All UniTope-tagged TCR-T cells performed equally well, with no significant reduction in functionality compared to untagged TCR-T cells. (In TRBC, P1 = A199, P2 = P198, P3 = L200, P4 = D202, P5 = A245, P6 = D238, P7 = W204; in TRAC P8 = D213, P9 = I209, P10 = P211, and P11= T214).

**Figure 7:**
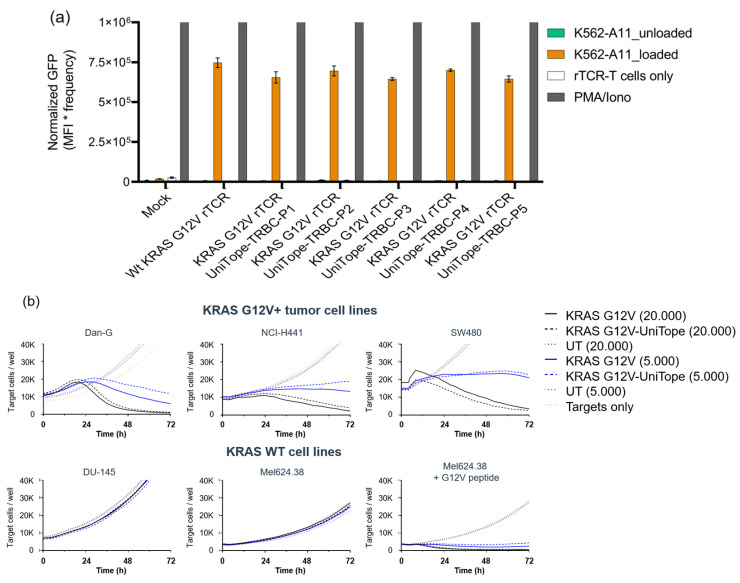
Functional validation of tagged versus untagged TCRs. (**a**) Functionality is demonstrated for one rTCR tagged at different positions with UniTope: TCR-Jurkat biosensor cells expressing rTCR tagged with UniTope were co-cultured with peptide-loaded K562-A11 cells or unloaded K562-A11 cells (control) (P1 = A199, P2 = W240, P3 = D202, P4 = A245, and P5 = I209 (TRAC)). Induced eGFP levels after 24 h of co-culture were determined by flow cytometry. (**b**) Lysis of target cells (red fluorescence) co-cultured with CD^8+^ rTCR-T cells: cytotoxicity assays using varying effectors to target cell ratios showed equivalent activity between tagged and untagged rTCRs. Red fluorescent object counts were quantified every 4 h for up to 72 h using the IncuCyte S3 live-cell imaging system. Kinetics of tumor cell killing are shown as reduction in NucLightRed+ object counts over time. Exemplary data are shown for one donor and one position.

Functionality was demonstrated for KRASG12V rTCRs tagged with UniTope inserted at different positions. Jurkat biosensor cells expressing tagged rTCRs were co-cultured with peptide-loaded T2 (Figure 6) or K562 (Figure 7a) cells, both modified to express HLA-A*11:01 (T2-A11 or K562-A11), for stimulation, using unloaded A11-positive cells as background controls. Induced eGFP levels after 24 h of co-culture were determined by flow cytometry. In this antigen-stimulation assay, tagged and untagged rTCRs induced comparable levels of eGFP in the rTCR-expressing Jurkat biosensor cells.

KRAS G12V-positive target cells are killed equally by CD8^+^ KRAS G12V-specific TCR-T cells with or without the UniTope-tag (Figure 7b). No differences were detectable with high (20,000) or low (5000) effector cell numbers. Non-specific killing of control cell lines (expressing KRAS WT) was not detected for either rTCR-T cell population.

### 3.6. UniTope Tagging Is Universal for rTCRs with Activity Comparable to Unmodified rTCRs

**Figure 8:**
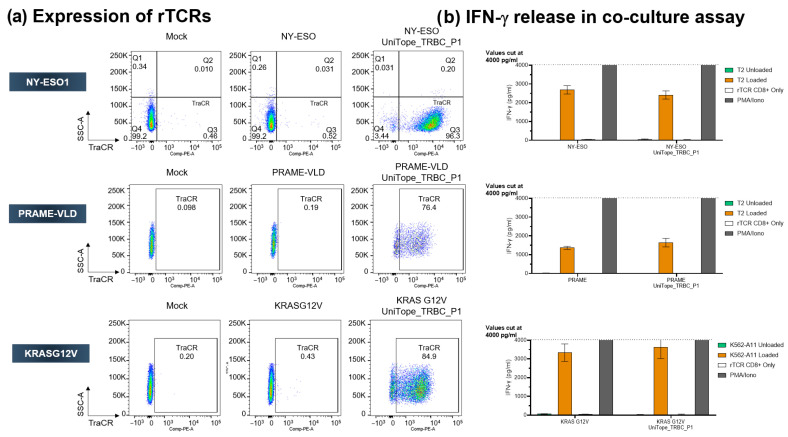
Universal applicability of UniTope in TCRs with different specificities. (**a**) FACS analysis of CD8^+^ T cells expressing tagged or untagged TCRs stained with fluorescent TraCR antibody. (**b**) Antigen-dependent IFN-γ secretion by engineered CD8^+^ T cells. IFN-γ was quantified after 24 h of co-culture of rTCR-transduced CD8^+^ T cells with peptide-pulsed T2 cells (effector/target ratio of 1:1).

To examine universal applicability of UniTope tagging for rTCRs, additional tagged rTCRs with different specificity were studied. Figure 8 shows the comparison of NY-ESO-1-, PRAME-, and KRASG12V-specific TCRS untagged or bearing the UniTope tag. These experiments showed that TraCR is specific for UniTope, since untagged TCR-T cells were not stained (Figure 8a). The three tagged rTCRs were expressed well at the cell surface (Figure 8a) and fully functional with respect to IFN-γ release upon antigen exposure (Figure 8b). The amount of IFN-γ secreted was comparable between T cells expressing UniTope-tagged rTCRs and untagged rTCRs. Non-specific activation of UniTope-tagged TCR-T cells was not observed. Two additional rTCRs were also assessed with comparable results (unpublished observations).

### 3.7. Analysis of Specificity and Cross-Reactivity

**Figure 9:**
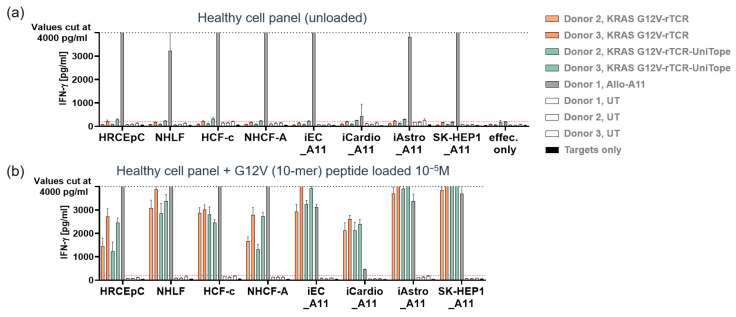
Healthy cell toxicity analysis. IFN-γ release by UniTope-tagged or untagged TCR-T cells after 24 h of co-culture with healthy cells. Co-culture supernatants of the indicated healthy cell types and tagged or untagged TCR-T cells were analyzed for IFN-γ by ELISA. (**a**) UniTope or untagged rTCR-T cells or untagged control T cells at 1 × 10^5^ cells per 96-well plate were co-cultured for 24 h with target cells (w/o peptide). (**b**) As positive controls, target cells were loaded with 10 µM of KRASG12V peptide. The number of target cells seeded per well was adapted for each cell type based on cell size, morphology, and growth kinetics. Healthy cells were co-cultured with TCR-T cells recognizing only HLA-A11 (Allo-A11) and were used as positive controls. Target cells without T cells (targets only) were included as negative controls. Depicted are mean values of triplicates with standard deviations. The dashed line indicates the highest IFN-γ standard of 4000 pg/mL, and values above this standard are displayed as an IFN-γ concentration of 4000 pg/mL. UT: untransduced T cells; UniTope- or untagged-TCR-T cells, KRASG12V-specific TCR-T cells; and Allo-A11 TCR-T cells, human renal cortical epithelial cells (HRCEpC), normal human lung fibroblasts (NHLF), human cardiac fibroblasts (HCF), iCell endothelial cells (iEC), iCell Cardiomyocytes (iCardio), iCell Astrocytes (iAstro), and SK-Hep-1 cell line (SK-Hep1). The index “_A11” indicates transduction of target cells with HLA-A*11.01.

Staining a diverse panel of cells representing healthy tissues, as described in [15], as well as PBMCs, yielded no signs of non-specific binding above the isotype control threshold (Figure 9). These results indicated minimal off-target risk of TraCR binding and suitability for translational use.

## 4. Discussion

The UniTope & TraCR System establishes a long-sought analytical bridge in rTCR engineering. The tag–antibody pair provides a universal, high-affinity, low-immunogenicity system applicable to diverse receptor scaffolds. Similar in concept to CAR “safety tags” such as RQR8 or E-tag [16,17], UniTope extends the principle to native heterodimeric TCRs, which are structurally more constrained. By embedding the tag in the constant region, rather than using external spacers, the native TCR geometry and signaling remain intact. This approach allowed for a precise definition of a universal analytical interface to be made.

Comprehensive functional testing demonstrated that insertion of the 12 aa UniTope sequence within the constant domain loop altered neither receptor assembly nor downstream signaling. Structural modelling confirmed that flanking Gly-Ser linkers alleviate local steric stress. This aligns with prior observations that short hydrophilic tags positioned in flexible loops can be tolerated without perturbing function [18].

The absence of any effect on cytokine secretion or cytotoxicity underscores the suitability of the tag for therapeutic constructs, including those intended for clinical use. Thereby, precise assessments established the sites for localization with functional neutrality and structural compatibility.

The TraCR monoclonal antibody supports multiple analytical modalities: flow cytometry, Western blot, immunoprecipitation, and potentially also ELISA-based quantification of soluble receptor fragments. This enables standardized quality testing from vector design through to studies of cell therapies for patient application. This single-reagent system can streamline batch comparability, confirmation of identity, and stability testing, when multiple patients are included in TCR-T cell clinical trials. The analytical versatility of the system provides enormous advantages for many phases of TCR-T therapy development and clinical application.

Small epitope tags, like FLAG (DYKDDDDK), 6×His, HA (YPYDVPDYA), c-Myc (EQKLISEEDL), and Strep-tag II (WSHPQFEK), are designed to be recognized by specific antibodies. Such tags can be inserted in positions described here (Supplementary Figure S1) but have deficits compared to UniTope. While often assumed to be “minimally” immunogenic, these sequences are antigenic enough to elicit specific antibody responses. Appropriate controls and potential tag removal may be required to interpret in vivo studies. Therefore, a close-to-human sequence tag, like UniTope, is better suited for use in clinical settings [7].

Although short tags can theoretically elicit immune responses, sequence alignment showed no significant homology of UniTope to human non-TCR-related or microbial proteins, minimizing risk for immunogenicity. Additionally, the strong homology of UniTope with human proteins (TRBV of the TCR, ATP1B3) strengthens the assumption of low immunogenicity. Nevertheless, clinical translation will require immunogenicity testing in human PBMC assays and humanized mice. Incorporation of human-derived motif variants could further reduce potential recognition by patient antibodies.

Beyond TCR-T cells, UniTope tagging could facilitate detection of other multichain immune receptors like B cell receptors, NK cell receptors, or synthetic signaling complexes, such as TCR-based T cell engagers or other hybrid receptor formats. Current alternatives include FLAG, HA, or V5 tags and truncated cell-surface markers (e.g., LNGFR and CD34) [19,20,21,22,23,24,25,26,27,28,29,30,31]. However, these usually require long linkers, risk immunogenicity, or interfere with receptor folding. Additionally, these tags need additional reagents to confirm detection of the product and to make distinctions to other cells, such as cells naturally expressing a CD34-positive stem and progenitor cells [24,32]. The compact UniTope epitope, fully integrated in a rTCR sequence, avoids these drawbacks. Moreover, the availability of a dedicated monoclonal antibody with exclusive specificity for the universal tag, eliminates variability associated with commercial reagents that can change between batches. The present work primarily evaluated in vitro expression and the function of αβ rTCRs, experiments with γδ TCRs, and single-chain TCRs or CARs remain to be investigated. Future in vivo studies should assess persistence and immunogenicity in animal models, as well as compatibility with downstream clinical assays, such as immunohistochemistry on patient biopsies. Nonetheless, the robustness and specificity demonstrated here provide a strong rationale for translational advancement of the UniTope & TraCR System.

## 5. Conclusions

We have developed and validated a universal tagging system for engineered T cell receptors comprising a compact peptide tag (UniTope) and its high-affinity antibody (TraCR). This system enables standardized quantification, visualization, and quality control of rTCRs, without compromising receptor function.

The UniTope technology represents a modular analytical interface poised to enhance reproducibility, regulatory compliance, and comparability across TCR-based therapeutic programs. Its expansion into multiplex and orthogonal tagging schemes further broadens applicability to complex cell therapy designs.

## Data Availability

The original contributions presented in this study are included in the article/Supplementary Material. Further inquiries can be directed to the corresponding author.

## Supplementary Materials

The following supporting information can be downloaded at https://www.mdpi.com/article/10.3390/medsci14010018/s1. Figure S1: FACS analysis of Jurkat biosensor cells expressing tagged or untagged rTCRs stained with fluorescent mAb specific for TRBV5-5.

## Abbreviations

The following abbreviations are used in this manuscript:

3D	Three-dimensional
aa	Amino acid
AI	Artificial intelligence
ELISA	Enzyme-linked immunosorbent assay
HRP	Horse radish peroxidase
KRAS	Kirsten rat sarcoma viral oncogene homolog
MFI	Median fluorescence intensity
NY-ESO-1	New York Esophageal Squamous Cell Carcinoma-1
OD	Optical density
PBMC	Peripheral blood mononuclear cell
pMHC	peptide–MHC
PRAME	Preferentially expressed antigen in melanoma
rTCR	Recombinant T cell receptor
TCR	T cell receptor
TRAC	TCR α constant regions
TRBC	TCR β constant regions
TRBV	TCR β variable regions
UT	Untransduced

## References

[1] June C.H., Sadelain M. (2018). Chimeric antigen receptor therapy. N. Engl. J. Med..

[2] Shafer P., Kelly L.M., Hoyos V. (2022). Cancer Therapy With TCR-Engineered T Cells: Current Strategies; Challenges; and Prospects. Front. Immunol..

[3] Stromnes I.M., Schmitt T.M., Chapuis A.G., Hingorani S.R., Greenberg P.D. (2014). Re-adapting T cells for cancer therapy: From mouse models to clinical trials. Immunol. Rev..

[4] Malviya M., Aretz Z.E.H., Molvi Z., Lee J., Pierre S., Wallisch P., Dao T., Scheinberg D.A. (2023). Challenges and solutions for therapeutic TCR-based agents. Immunol. Rev..

[5] Rath J.A., Arber C. (2020). Engineering Strategies to Enhance TCR-Based Adoptive T Cell Therapy. Cells.

[6] Knabel M., Franz T.J., Schiemann M., Wulf A., Villmow B., Schmidt B., Bernhard H., Wagner H., Busch D.H. (2002). Reversible MHC multimer staining for functional isolation of T-cell populations and effective adoptive transfer. Nat. Med..

[7] Scarfe J., Kosmützky D., Nisbet R.E.R. (2025). A game of tag: A review of protein tags for the successful detection, purification and fluorescence labelling of proteins expressed in microalgae. Plant J..

[8] Haryadi R., Ho S., Kok Y.J., Pu H.X., Zheng L., Pereira N.A., Li B., Bi X., Goh L.-T., Yang Y. (2019). Optimization of heavy chain and light chain signal peptides for high level expression of therapeutic antibodies in CHO cells. PLoS ONE.

[9] Bürdek M., Prinz P.U., Mutze K., Tippmer S., Geiger C., Longinotti G., Schendel D.J. (2025). Characterization of a 3S PRAME VLD-Specific T Cell Receptor and Its Use in Investigational Medicinal Products for TCR-T Therapy of Patients with Myeloid Malignancies. Cancers.

[10] Coluccio A., Tippmer S., Prinz P., Buerdek M., Mutze K., Loesch B., Davari K., Longinotti G., Schendel D.J. (2023). T cells co-expressing a highly potent NY-ESO-1-specific TCR and a chimeric PD1-41BB co-stimulatory switch receptor show a favorable polyfunctional profile for the treatment of solid tumors. Cancer Res..

[11] Crame K., Longinotti G., Catarinella M., Prinz P., Tippmer S., Mutze K., Coluccio A., Salvermoser M., Bittmann J., Bürdek M. (2023). Mitigation of tumor microenvironment-mediated immunosuppression using a PD1-41BB switch protein with optimal affinity TCRs for first-in-class, 3rd generation TCR-T therapies. Ann. Oncol..

[12] Chiampanichayakul S., Khunkaewla P., Pata S., Kasinrerk W. (2006). Na, K ATPase beta3 subunit (CD298): Association with alpha subunit and expression on peripheral blood cells. Tissue Antigens.

[13] LaCroix-Fralish M.L., Mo G., Smith S.B., Sotocinal S.G., Ritchie J., Austin J.-S., Melmed K., Schorscher-Petcu A., Laferriere A.C., Lee T.H. (2009). The beta3 subunit of the Na+, K+-ATPase mediates variable nociceptive sensitivity in the formalin test. Pain.

[14] Zhang S.H., Liu D.-X., Wang L., Li Y.-H., Wang Y.-H., Zhang H., Su Z.-K., Fang W.-G., Qin X.-X., Shang D.-S. (2019). A CASPR1-ATP1B3 protein interaction modulates plasma membrane localization of Na+/K+-ATPase in brain microvascular endothelial cells. J. Biol. Chem..

[15] Bürdek M., Prinz P.U., Mutze K., Bosch M., Tippmer S., Coluccio A., Geiger C., Majumder S., Longinotti G., Schendel D.J. (2025). Preclinical development of costimulatory switch protein (CSP)-armored NY-ESO-1/LAGE-1a-specific TCR-T cells for therapy of hard-to-treat PD-L1-positive solid tumors. Int. J. Transl. Med..

[16] Mosti L., Langner L.M., Chmielewski K.O., Arbuthnot P., Alzubi J., Cathomen T. (2021). Targeted multi-epitope switching enables straightforward positive/negative selection of CAR T cells. Gene Ther..

[17] Arakawa T., Akuta T. (2025). Beyond Purification: Evolving Roles of Fusion Tags in Biotechnology. Curr. Issues Mol. Biol..

[18] Ashmore-Harris C., Iafrate M., Saleem A., Fruhwirth G.O. (2020). Non-invasive Reporter Gene Imaging of Cell Therapies; including T Cells and Stem Cells. Mol. Ther..

[19] Terpe K. (2003). Overview of tag protein fusions: From molecular and biochemical fundamentals to commercial systems. Appl. Microbiol. Biotechnol..

[20] Götzke H., Kilisch M., Martínez-Carranza M., Sograte-Idrissi S., Rajavel A., Schlichthaerle T., Engels N., Jungmann R., Stenmark P., Opazo F. (2019). The ALFA-tag is a highly versatile tool for nanobody-based bioscience applications. Nat. Commun..

[21] Bister A., Ibach T., Haist C., Gerhorst G., Smorra D., Soldierer M., Roellecke K., Wagenmann M., Scheckenbach K., Gattermann N. (2022). Optimized NGFR-derived hinges for rapid and efficient enrichment and detection of CAR T cells in vitro and in vivo. Mol. Ther. Oncolytics.

[22] Lichty J.J., Malecki J.L., Agnew H.D., Michelson-Horowitz D.J., Tan S. (2005). Comparison of affinity tags for protein purification. Protein Expr. Purif..

[23] Bister A., Ibach T., Haist C., Smorra D., Roellecke K., Wagenmann M., Scheckenbach K., Gattermann N., Wiek C., Hanenberg H. (2021). A novel CD34-derived hinge for rapid and efficient detection and enrichment of CAR T cells. Mol. Ther. Oncolytics.

[24] Norell H., Zhang Y., McCracken J., Martins da Palma T., Lesher A., Liu Y., Roszkowski J.J., Temple A., Callender G.G., Clay T. (2010). CD34-based enrichment of genetically engineered human T cells for clinical use results in dramatically enhanced tumor targeting. Cancer Immunol. Immunother..

[25] Fritschle K., Mielke M., Seelbach O.J., Mühlthaler U., Živanić M., Bozoglu T., Dötsch S., Warmuth L., Busch D.H., Skerra A. (2025). The V5-Epitope Tag for Cell Engineering and Its Use in Immunohistochemistry and Quantitative Flow Cytometry. Biology.

[26] Kandarakov O.F., Polyakova N.S., Petrovskaya A.V., Bruter A.V., Belyavsky A.V. (2024). CD52/FLAG and CD52/HA Fusion Proteins as Novel Magnetic Cell Selection Markers. Int. J. Mol. Sci..

[27] Fehse B., Richters A., Putimtseva-Scharf K., Klump H., Li Z., Ostertag W., Zander A.R., Baum C. (2000). CD34 splice variant: An attractive marker for selection of gene-modified cells. Mol. Ther..

[28] Einhauer A., Jungbauer A. (2001). The FLAG peptide; a versatile fusion tag for the purification of recombinant proteins. J. Chromatogr. B.

[29] Kimple M.E., Brill A.L., Pasker R.L. (2013). Overview of affinity tags for protein purification. Curr. Protoc. Protein Sci..

[30] Abcam Epitope Tags: HA (Human Influenza Hemagglutinin) Tag Derived Sequence and Usage. https://www.abcam.com/en-us/technical-resources/guides/fusion-tags-guide/epitope-tags.

[31] Casucci M., Falcone L., Camisa B., Norelli M., Porcellini S., Stornaiuolo A., Ciceri F., Traversari C., Bordignon C., Bonini C. (2018). Extracellular NGFR spacers allow efficient tracking and enrichment of fully functional CAR-T cells co-expressing a suicide gene. Front. Immunol..

[32] Sidney L.E., Branch M.J., Dunphy S.E., Dua H.S., Hopkinson A. (2014). Concise review: Evidence for CD34 as a common marker for diverse progenitors. Stem Cells.

