# Supplementary material for: UniTope & TraCR: A Universal Tool to Tag, Enrich, and Track TCR-T Cells and Therapeutic Proteins
Source: Med Sci (Basel). 2025 Dec 31;14(1):18. doi: 10.3390/medsci14010018 (PMC12821693; doi:10.3390/medsci14010018)
Supplement: Supplementary file 1 [file medsci-14-00018-s001.zip › medsci-4038591-supplementary.pdf]

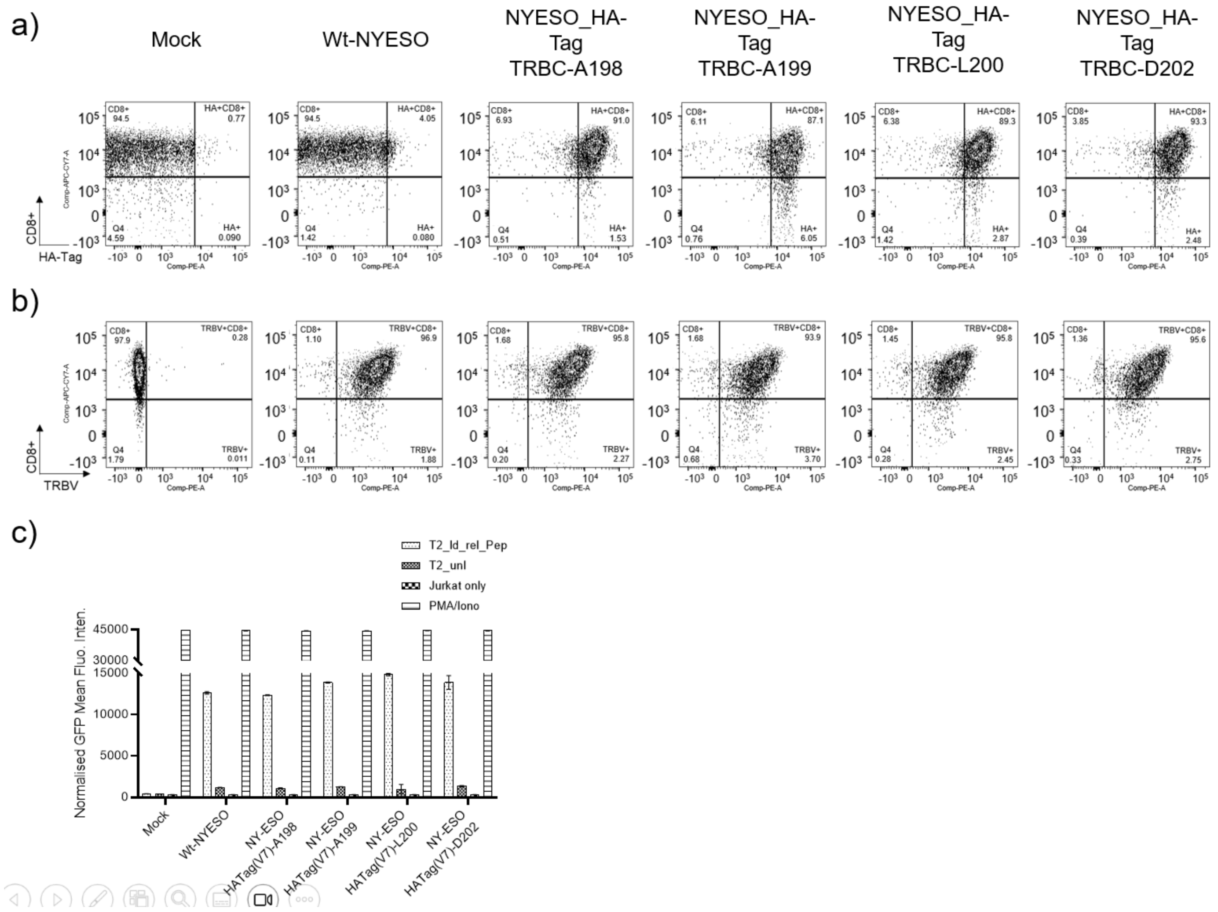

Supplementary Figure 1: FACS analysis of Jurkat biosensor cells expressing tagged or untagged rTCRs stained with fluorescent mAb specific for TRBV5-5 (a) or HA-tag specific antibody (b). The columns show mock transduced, untagged rTCRs as well as rTCRs tagged with HA-tag at different positions. (c) To verify correct functionality, Jurkat biosensor cells were co-cultured with T2 cells pulsed with (loaded) or without (unloaded) relevant peptide, along with controls (negative control: rTCR-expressing Jurkat biosensor cells only; positive control for full activation: PMA/Ionomycin). After 24 h, the eGFP signal was analyzed by flow cytometry as the read-out for rTCR activation. All HA-tagged TCR-T cells performed equally well, with no significant reduction in functionality compared to untagged TCR-T cells. This confirms that the chosen positions in the constant region of the rTCR are functionally inert and can be used with different tags.
